# Alcoholism

**Published:** 1903-09-26

**Authors:** 


### ALCOHOLISM.

Morbid conditions due to alcoholism are among the commonest of those with which medical men have to deal, and their treatment is often unsuccessful. According to Dr. Crothers,[1] this failure is due to two main causes, one being that the connection of such cases with alcoholism is often not recognised at all, while the other is that even when alcoholism is duly recognised as the cause, the treatment is based upon a false conception of the true pathology of this disease. Dr. Crothers appears to think that the introduction by Sutton of the term delirium tremens in the early part of the nineteenth century is partly responsible for this obscuration, since it brought into prominence one result of alcoholism which has marked and peculiar characteristics, but which should not be taken as the leading type of alcoholic disease and which is not in itself nearly so common as the familiar use of the term would imply. The characteristic symptoms of this condition are muscular tremors and a special class of mental and visual delusion, and when it comes on in the absence of a precipitating cause such as an accident or acute illness, it is a proof that indulgence in alcohol has produced definite pathological lesions, and the prognosis is always more or less grave. Many cases, however, which are diagnosed as delirium tremens by individual observers are not of this dangerous character, so far as the alcoholic element is concerned, the true cause being shock from injury or the toxins produced by acute specific or inflammatory disorder. According to Dr. Crothers, all forms of alcoholism are toxæmias pure and simple, whether the immediate cause be a few glasses of unaccustomed drink or many months of continuous dependence on stimulants. Midway between the delirium tremens which has an accident or constitutional disorder for its precipitating cause and the attacks which come on so to speak *sponte suâ* are those cases in which the more obvious and striking symptoms have not developed, and in which there is merely a tendency towards confusion of thought and an incapacity for prolonged concentration of mind which finds its physical analogue in a tremulous tongue and quivering hand. This again is due to toxæmia of the same general character as that to which the acute symptoms of typical delirium tremens are due, but is demonstrative of less complete pathological change. As for the treatment of admitted alcoholism, Dr. Crothers is opposed to the custom of slowly stopping the imbibition of alcohol with a view to the avoidance of shock. There is no shock caused by the stoppage of other poisons, so why, he asks, should shock be apprehended in the case of alcohol. In his opinion it should be withheld entirely and at once. The next step is to produce sharp purgation and free sweating. Until free purgation and free perspiration have been established for some little time he is opposed to the administration of any drug of a narcotic order, and in the primary stage gives the smallest possible quantity of food. The acute stage over, insomnia frequently occurs and may call for the exhibition of the milder sedatives, of which chloretone has proved the most satisfactory in his hands. Its action does not appear to be in any way cumulative, nor in effective doses of 15 and 20 grains does it appear to cause any interference with nutrition. It is best employed in the evening. An hypnotic action is usually evident in half an hour, but even in default of actual sleep there is a general tranquility and dreamy relaxation which is even more desirable than the action of the direct and powerful narcotic drugs. Its use may be continued as long as necessary, and as a means of meeting the alcoholic yearning a strong infusion of quassia is very effective. To complete a cure prolonged care is necessary, and much attention to feeding and the promotion of nutrition required. Of tonic remedies cinchona is the best, but arsenic and phosphoric acid are both useful. All other remedies, however, should be subordinate to the continuation of means and measures which act on the skin and encourage elimination.

[1] American Medicine, July 26.

